# When Corporate Social Responsibility (CSR) Meets Organizational Psychology: New Frontiers in Micro-CSR Research, and Fulfilling a Quid Pro Quo through Multilevel Insights

**DOI:** 10.3389/fpsyg.2017.00520

**Published:** 2017-04-07

**Authors:** David A. Jones, Chelsea R. Willness, Ante Glavas

**Affiliations:** ^1^Grossman School of Business, University of Vermont, BurlingtonVT, USA; ^2^Edwards School of Business, University of Saskatchewan, SaskatoonSK, Canada; ^3^Department of Corporate Social Responsibility, Strategy and Entrepreneurship, KEDGE Business SchoolMarseille, France

**Keywords:** corporate social responsibility, corporate social performance, sustainability, organizational psychology, microfoundations, multilevel theory, micro-CSR, stakeholder

## Abstract

Researchers, corporate leaders, and other stakeholders have shown increasing interest in Corporate Social Responsibility (CSR)—a company’s discretionary actions and policies that appear to advance societal well-being beyond its immediate financial interests and legal requirements. Spanning decades of research activity, the scholarly literature on CSR has been dominated by meso- and macro-level perspectives, such as studies within corporate strategy that examine relationships between firm-level indicators of social/environmental performance and corporate financial performance. In recent years, however, there has been an explosion of micro-oriented CSR research conducted at the individual level of analysis, especially with respect to studies on how and why job seekers and employees perceive and react to CSR practices. This micro-level focus is reflected in 12 articles published as a Research Topic collection in *Frontiers in Psychology* (Organizational Psychology Specialty Section) titled “CSR and organizational psychology: Quid pro quo.” In the present article, the authors summarize and integrate findings from these Research Topic articles. After describing some of the “new frontiers” these articles explore and create, the authors strive to fulfill a “quid pro quo” with some of the meso- and macro-oriented CSR literatures that paved the way for micro-CSR research. Specifically, the authors draw on insights from the Research Topic articles to inform a multilevel model that offers multiple illustrations of how micro-level processes among individual stakeholders can explain variability in meso (firm)-level relationships between CSR practices and corporate performance. The authors also explore an important implication of these multilevel processes for macro-level societal impact.

## Introduction

For-profit companies are increasingly focused on managing how internal and external stakeholders perceive and react to business practices pertaining to Corporate Social Responsibility (CSR)—a company’s discretionary actions, policies, and programs that appear to advance societal well-being in ways that extend beyond its immediate financial interests and the requirements of the law (McWilliams and Siegel, 2001). CSR initiatives are usually designed to take into account stakeholder expectations about the triple bottom line of economic, social, and environmental performance (Aguinis, 2011).

Since the early 1950s when the CSR concept came to fruition, much of the associated scholarly research has been dominated by a “macro focus that emphasized broad firm-wide policies, thereby laying the responsibility for attaining CSR results directly on top-level managers and the overall strategies they adopted” (Frederick, 2016, p. 2). This firm-level focus in CSR research has been described as a “macro” perspective by Frederick and others, whereas researchers in some disciplines would describe it as a “meso” perspective. For clarity, we adopt the labels and distinctions between three levels of analysis described by Frynas and Stephens (2015, p. 485): “the micro level (involving psychological bases among individuals), the meso level (involving relational issues among organizations), and the macro level (involving wider political, economic and societal dynamics)”^1^. As Frederick (2016) observed, much of the broader CSR literature comprises meso-level theory and research, including a number of studies conducted by business strategy scholars who strive to understand relationships between firm-level CSR practices (or corporate social performance) and indicators of firm-level performance, including corporate financial performance (e.g., Waddock and Graves, 1997; Orlitzky et al., 2003). Macro-level research has also advanced the science and practice of CSR by highlighting how CSR phenomena are shaped by the broader economic, institutional, political, and societal contexts in which they are embedded (e.g., Matten and Moon, 2008; Frynas and Stephens, 2015). In contrast to the amount of meso- and macro-level CSR research, relatively few micro-level studies exist (Aguinis and Glavas, 2012), which has left a gap in the scholarly understanding of the intersections between a company’s CSR practices, the broader contexts in which they are embedded, and the associated experiences and reactions among the company’s own people.

Employees, as well as prospective employees, are important stakeholders who both influence and are influenced by an employer’s CSR practices (McWilliams and Siegel, 2001; Aguilera et al., 2007). In some cases, specific CSR initiatives and programs are created by a company’s employees, and in most cases a company’s employees are involved in implementing its CSR practices. Moreover, a growing body of empirical evidence shows that the decisions and behaviors of job seekers and employees create meaningful demand for companies to invest in substantive CSR practices (Jones and Rupp, in press). In hindsight, the lack of attention paid by CSR scholars to a firm’s internal stakeholders is somewhat surprising, to say the least. But this knowledge gap is now being filled by what can be appropriately described as an explosion of micro-CSR research conducted at the individual-level of analysis focusing on how and why job seekers, employees, and other individuals perceive and react to CSR (Glavas, 2016a). This explosion of scholarly activity is reflected in the recent publication of several reviews of micro-CSR research on employee recruitment, and reactions to CSR among incumbent employees (Peloza and Shang, 2011; Aguinis and Glavas, 2012; Jones and Willness, 2013; Willness and Jones, 2013; Rupp and Mallory, 2015; Glavas, 2016b; Gond et al., 2017; Jones and Rupp, in press). The micro-CSR literature also appears to be undergoing rapid maturation, as evidenced by advances in measurement (El Akremi et al., 2015), and the development of overarching theories about how and why individuals react to CSR practices, including theory about employee motives (Rupp et al., 2006; Bauman and Skitka, 2012; Glavas, 2016b; Jones and Rupp, in press), the underlying mechanisms through which job seekers are attracted by CSR (Jones et al., 2014), and a needs-based model of CSR motives that applies to micro-, meso-, and macro-level stakeholders (Aguilera et al., 2007).

In this context, we sought to promote new advances to micro-CSR theory and research by co-editing a *Frontiers in Psychology* (Organizational Psychology Specialty Section) Research Topic collection titled “CSR and organizational psychology: Quid pro quo.” In the present article, we describe the diversity of perspectives and approaches applied in the 12 Research Topic articles, and some of the “new frontiers” these articles explore and create for micro-CSR theory and research. We then draw on insights from these Research Topic articles in an effort to fulfill a “quid pro quo” with some of the meso- and macro-oriented literatures that paved the way for micro-CSR scholarship. Specifically, we provide a number of examples grounded in the Research Topic articles that can inform multilevel conceptualizations of CSR phenomena, focusing on how micro-level processes among individual stakeholders can explain variability in meso (firm)-level relationships between CSR practices and corporate performance. We also consider an important implication of these multilevel processes for macro-level societal impact.

## A Diversity of Approaches and Perspectives in the 12 Micro-CSR Research Topic Articles

This collection of 12 Research Topic articles illustrates a rich diversity of conceptual and methodological approaches used to advance the micro-CSR literature, the kinds of stakeholders on which these studies focus, and the types of CSR practices examined. With respect to the diversity of conceptual and methodological approaches, these Research Topic articles include a commentary on the history of macro-, meso-, and micro-CSR research (Frederick, 2016), a literature review of micro-CSR theory and research (Glavas, 2016a), conceptually driven theoretical development (Voliotis et al., 2016), experimental research utilizing quantitative and qualitative data (Bridoux et al., 2016; Jones et al., 2016), an assessment of interview data (Seivwright and Unsworth, 2016), an intervention study (Russell et al., 2016), survey-based field research (Glavas, 2016b; Hameed et al., 2016; Jones, 2016; Unsworth et al., 2016), and a meta-analysis (Wiernik et al., 2016).

Most of the empirical studies among these Research Topic articles focus on *employees* as the focal stakeholder group, including studies of how employees conceptualize their employer’s responsible or irresponsible practices (Seivwright and Unsworth, 2016; Voliotis et al., 2016), how they respond to CSR practices (Glavas, 2016b; Hameed et al., 2016; Jones, 2016), and their work behaviors that contribute to their employer’s CSR initiatives (Russell et al., 2016; Seivwright and Unsworth, 2016; Wiernik et al., 2016). Other Research Topic articles focus on other individual-level stakeholders, including job seekers (Bridoux et al., 2016; Jones et al., 2016), customers (Bridoux et al., 2016), and members of the general public (Unsworth et al., 2016). In addition to this diversity of stakeholders, these articles also vary in the types of CSR practices examined. Some articles focus on the intersection of business practices and climate change (Unsworth et al., 2016) and companies’ environmental practices (Russell et al., 2016; Wiernik et al., 2016), whereas other articles focus on employee volunteerism (Jones, 2016) or both community involvement and environmentally sustainable business practices (Jones et al., 2016). Another article focuses on the distinction between internal vs. external CSR practices (Hameed et al., 2016), and other articles focus on multiple types of CSR practices examined separately (Bridoux et al., 2016; Seivwright and Unsworth, 2016) or together as a broader CSR concept or composite (Frederick, 2016; Glavas, 2016a,b; Voliotis et al., 2016). These Research Topic articles also include critical analyses and empirical tests of the veracity of beliefs and assumptions held by societal members and scholars alike (Jones, 2016; Wiernik et al., 2016), and explicit considerations of contexts in which stakeholders react negatively to well-intentioned CSR practices (Jones et al., 2016) and to social irresponsible practices (Voliotis et al., 2016). Together, the Research Topic articles offer a diversity of conceptual and methodological approaches that can be used to study micro-CSR topics, and they highlight a variety of stakeholders and CSR practices on which future researchers can focus in isolation or in combination.

We now turn to describing “new frontiers” for micro-CSR theory and research based on insights and findings from the 12 Research Topic articles. We begin by summarizing key points from the two commentary and literature review articles, followed by describing some of the “new frontiers” illuminated by the remaining ten articles that we discuss in alphabetical order by the authors’ last names. In highlighting these “new frontiers,” our purpose is to illuminate some of the valuable insights that can be gleaned from reading each Research Topic article, and to emphasize new directions for micro-CSR theory and research.

## “New Frontiers” Explored and Created by the 12 Research Topic Articles

In this brief but impactful commentary, renowned CSR scholar William Frederick expands upon an article that he wrote in 2008 through his Research Topic article titled *Corporate social responsibility: Deep roots, flourishing growth, promising future*. The commentary provides an excellent frame for the other articles in this Research Topic collection by describing macro, meso, and micro CSR and advocating for integration between the levels into a holistic analysis of CSR. Frederick (2016) also offers an interesting historical snapshot of the concept of CSR, and its evolution from the early 1950s to present day. He closes with an urgent call to action in the context of climate change and global environmental challenges to create a coalition between the policy makers and the people. Further, he characterizes the articles in this Research Topic as an effective starting place for conversations and new ideas about how to attain the “Policy to People” goal that he suggests is so critically needed.

The collection of Research Topic articles also includes a review of micro-CSR theory and research, titled *Corporate social responsibility and organizational psychology: An integrative review.* In this article, Glavas (2016a) reviews relevant work across 166 articles, book chapters, and books, and he highlights potential synergies between organizational psychology and CSR that create opportunities to advance the broader CSR literature. For instance, while micro-CSR research on employees has focused on the outcomes of employee beliefs and perceptions of CSR practices (i.e., employee responses to CSR), there has been relatively less emphasis on understanding the underlying mechanisms (i.e., the psychological mechanisms that mediate CSR-employee outcome relationships). Organizational and applied psychology, Glavas points out, have a rich history of theoretical development that has been used to understand underlying mechanisms. He reviews extant applications of theories in micro-CSR research that include organizational justice, social exchange, ethics, values alignment, and individual differences. The author also encourages “new frontiers” by proposing five areas for future research grounded in theories of organizational psychology, such as studies focusing on the intersection of CSR with work meaningfulness and an employee’s ability to realize his or her whole/ideal self at work. The underlying theme of his literature review also represents a “new frontier” through his more general assertion that CSR practices can be embedded in organizational designs and processes to make organizations more humanistic in nature.

“New frontiers” are also explored in an experimental study described in an article titled *Stakeholders’ responses to CSR tradeoffs: When other-orientation and trust trump material self-interest* by Bridoux et al. (2016). These authors focus on the theoretically and practically important topic of stakeholder reactions to CSR tradeoffs, which refers to a firm’s unbalanced allocation of resources to support CSR initiatives intended to benefit specific stakeholder groups. For instance, when a firm has relatively strong CSR practices with respect to its treatment of suppliers while having relatively weak CSR practices toward its own employees, how might customers or prospective employees respond? Might stakeholder responses be shaped by whether their own stakeholder group is the affected party at the favorable vs. unfavorable end of a CSR tradeoff? Using a sample of over 900 participants, these authors conducted a vignette-based experimental study to explore these questions and other novel theoretically grounded hypotheses across multiple contexts and scenarios. Their study results debunk the myth of the so-called “rational man” who reliably acts in the service of his or her own self-interest, by showing that people did not systematically respond more positively to a CSR tradeoff that favored their own group over another stakeholder group. Rather, the results paint a nuanced picture of stakeholder responses to CSR tradeoffs based on the interplay between whether a tradeoff favors one’s own or some other group, individual differences (i.e., other orientation), and organizational trust as a potential mediator that explains responses from two stakeholder groups: customers’ purchasing intentions and job seekers’ job pursuit intentions. In practice, CSR trade-offs are more likely to be the norm than the exception, given the multitude and diversity of pressures affecting managerial decisions about CSR-directed resource allocations, such as the competitive, economic, cultural, regulatory, and other institutional pressures faced by each company. As such, this article opens the door to “new frontiers” for micro CSR scholars who are well-equipped to conduct research and develop theory to explain stakeholder reactions to such CSR trade-offs.

In an article by Glavas (2016b) titled *Corporate social responsibility and employee engagement: Enabling employees to employ more of their whole selves at work*, the author builds on engagement theory in his investigation of whether CSR can enable employees to bring more of their whole selves to work and, as a result, be more engaged. Specifically, he tests two mediators through which CSR was hypothesized to promote engagement: perceived organizational support (POS) and authenticity (i.e., being able to show one’s true self at work). Although prior micro-CSR research has examined the roles of POS and related constructs, little to no empirical attention has been paid to whether CSR might be a vehicle through which employees can bring more aspects of their whole selves to work. The results of this study, based on survey responses from over 15,000 employees of a professional services firm, open up “new frontiers” in at least three ways. First, the study represents a shift from the more common top-down focus on what an organization can give to employees to a bottom-up approach where CSR is conceptualized as providing the conditions in which employees are doing the giving (i.e., employees giving themselves to their employer and those served by its CSR). Second, while research has shown that engaging in CSR as an extra-role pursuit (e.g., employee volunteering) can have positive effects on employees in the short term, there are few longitudinal studies that inform whether higher levels of extra-role CSR involvement might have negative effects on employees, as reflected in their work engagement. Finally, study results illustrate the importance of testing multiple mediators within the same empirical models, which has rarely occurred in micro-CSR research. For instance, the nature of an indirect effect observed in micro-CSR research might differ depending on whether a given mediator is tested on its own vs. in models that include other mediators, and such a difference may have profound implications for how scholars explain the processes through which CSR leads to employee outcomes.

In another survey-based field study, Hameed et al. (2016) explore “new frontiers” that can meaningfully inform CSR theory and practice in their article titled *How do internal and external CSR affect employees’ organizational identification? A perspective from the group engagement model.* These authors grounded their hypotheses in an important distinction between employees’ perceptions of internal CSR practices directed toward the firm’s employees, vs. external CSR practices directed toward stakeholders outside the firm, such as suppliers and the community. The authors draw on the group engagement model from the organizational justice literature and social identity theory to develop hypotheses about how internal and external CSR practices related to employees’ organizational identification via different mechanisms: perceived internal respect and perceived external prestige, respectively. Using survey data from 414 employees working in five multinational firms in Pakistan, study results demonstrate the value of distinguishing between internal vs. external CSR practices in micro CSR research that seeks to understand employee responses to CSR practices. The authors also present evidence highlighting how the extent to which employees view their work as a “calling” rather than a “job” (i.e., their calling orientation) shapes the relationship between their perceptions of their employer’s CSR practices and their organizational identification.

In another field study reported in an article titled *Widely assumed but thinly tested: Do employee volunteers’ self-reported skill improvements reflect the nature of their volunteering experience?*, Jones (2016) grounds his research in a critical observation: A frequently touted benefit to firms that invest in corporate volunteering programs is that their employees develop work-related skills through volunteering while on “company time” (i.e., as part of their daily work). Jones observes, however, that this assumption has received little to no empirical scrutiny in the scholarly literature, and is instead accepted as “fact” based largely on anecdotal reports from corporate leaders and employee volunteers. Using data from 74 employee volunteers who completed a 10-week service apprenticeship managed by a U.S.-based non-profit called Citizen Schools, Jones explores “new frontiers” by testing novel hypotheses about the extent to which self-reported skill development reflects characteristics of the employee volunteers and their volunteering experiences as theory and common sense dictate if skill development truly occurs. For instance, he tested hypotheses about whether employee volunteers who report having more opportunities to practice each of 10 skills report significantly greater development in those skills (e.g., leadership, mentorship, motivating others, project management, providing feedback, public speaking, teamwork, and time management). Jones also tested hypotheses about the interaction between characteristics of the volunteering experience and the employee volunteers’ self-efficacy about their ability to improve their work-related skills. Jones discusses how the support found for some study hypotheses informs new directions for research and theory, and the design of volunteer experiences that benefit employers and employees, while creating value for the communities and causes they serve.

Focusing on responses to CSR among job seekers rather than employees, Jones et al. (2016) studied the reasons why many job seekers tend to be attracted to working for employers known for their community involvement and environmentally sustainable practices. As suggested by the title of their article *Illuminating the signals job seekers receive from an employer’s community involvement and environmental sustainability practices: Insights into why most job seekers are attracted, others are indifferent, and a few are repelled*, study results also point to reasons why such practices can sometimes be ineffective, and even counterproductive. These authors conducted a substantive replication of prior support that was found for three signal-based mechanisms (Jones et al., 2014) by content analyzing written responses to two general questions about whether and why participants were (or were not) attracted to a target employer, and their impressions about the content of one of its webpages that included information about either of the two types of CSR practices examined. Their findings provided support for two previously established mechanisms, and extended prior work by identifying other signal-based mechanisms that might plausibly affect job seekers’ attraction to CSR (e.g., inferences about the characteristics of the company’s employees). Their study also creates “new frontiers” by exploring data that offers the first ever empirically driven insights into why some people are unaffected by an employer’s CSR practices, and a few might even be “turned off” by them (e.g., people’s skepticism and cynicism about the CSR practices).

Russell et al. (2016) tested the effects of an intervention on employees’ CSR-related behaviors in an article titled *Turn it off: An action research study of top management influence on energy conservation in the workplace.* These authors explore “new frontiers” by looking inside the organization to understand how the visibility of top management commitment to environmental practices through modeling and prompts/reminders might influence employees’ energy conservation behaviors. For instance, although researchers have explored the effects of prompts/reminders on environmental behaviors at home, this topic has received little to no attention in workplace settings. Deriving hypotheses from behavior change theory, the authors tested the effects of a three-pronged intervention (visual modeling, communication, and prompts) among employees of an Australian hospital using a pre–post-intervention design that included post-intervention measures taken 1 and 6 months later. Study data included objective measures of energy conservation (e.g., use of lights, and turning off computers and monitors) and subjective measures of the same variables, plus attitudinal measures like the degree to which participants felt energy conservation was part of the organizational culture and norms, and perceived commitment to such practices among top management. Study results provided general support for the efficacy of the intervention, including effects that were observed 6 months later, while some of the more nuanced findings open several “new frontiers” for researchers to explore (e.g., do the effects of the intervention weaken over time as employees become habituated to the prompts?).

In another Research Topic article titled *Making sense of corporate social responsibility at work*, Seivwright and Unsworth (2016) argue that in order to fully understand what influences employees’ engagement in CSR, it is critical to first understand how the employees themselves conceptualize CSR and its relation to their work. The authors note that CSR is often enacted or implemented by an organization’s employees, yet there has been comparatively little focus on how they understand CSR and how they contribute to it. The authors also distinguish between employees in non-profit vs. for-profit organizations, which may have important implications in terms of their perceptions and experiences. Using an exploratory inductive approach, Seivwright and Unsworth conducted semi-structured interviews with 32 employees, gathering data from both types of organizations (i.e., non-profit and for-profit). They asked employees about any instances of their CSR-related behavior at work, as well as why they engaged in that action, whether it was encouraged by the organization, any perceived obstacles, and how the CSR behavior made them feel. The results of this study showed important contrasts in how employees from non-profit vs. for-profit organizations conceptualized and engaged in CSR, especially regarding how CSR contributes to their experience of meaningfulness at work and in work. This article paves the way for several “new frontiers” including examining a more fulsome scope of behaviors that employees believe are part of CSR, comparing the perceptions of employees in organizations with arguably different roles and missions in society, and the implications of embedded vs. peripheral CSR (at the organizational strategy *and* individual job levels) on the experience of meaningfulness at work.

In an article on societal attitudes toward the role of business in combatting climate change, *Is dealing with climate change a corporation’s responsibility? A social contract perspective*, Unsworth et al. (2016) examine attitudes about anthropogenic climate change and free market ideology, and how this impacts people’s beliefs about the actors responsible for dealing with climate change (e.g., corporations, governments, local authorities, environmental groups, etc.). In their survey study of 1066 individuals across Australia, the authors examine the social contract aspects of CSR in terms of whether citizens believe companies have a legal responsibility to address climate change, and the factors that impact support for regulatory policy to act upon that belief. Their findings highlight “new frontiers” not only in research (e.g., micro-level data with societal-level implications) but also important repercussions for policy-makers, particularly given the prominent role of free market ideology in the pattern of effects and its potential to create barriers to change.

In a conceptual and theory development piece, *Perception-induced effects of corporate social irresponsibility (CSiR) for stereotypical and admired firms*, Voliotis et al. (2016) explore “new frontiers” by developing a model of how stakeholders react to corporate social irresponsibility (CSiR). Prior research has tended to focus on positive stakeholder reactions to CSR, whether those CSR practices are meaningful in scope and embedded in a firm’s operations, or more symbolic and superficial. Relatively overlooked, however, are negative stakeholder reactions to a firm’s irresponsible business practices. In another departure from existing perspectives in which CSR and CSiR are typically viewed as opposite ends of a single continuum, these authors propose there are distinct psychological mechanisms involved in interpreting and reacting to CSR vs. CSiR. Building on theories from the stereotype content model and the BIAS map (behaviors from intergroup affect and stereotypes), the authors propose a model to explain stakeholder reactions to CSiR. For a typical for-profit firm, the authors propose that stakeholders will react to CSiR through the mechanism of respect/disrespect and like/dislike, which can lead to anger and in turn motives to create harm for the firm. In contrast, for stakeholder reactions to CSiR among admired firms, the positive reputations of those firms can buffer stakeholders’ negative reactions. The authors explore other “new frontiers” by proposing various boundary conditions for these effects (e.g., perception of the firm’s culpability in the CSiR).

In Wiernik et al.’s (2016) article titled *Age and employee green behaviors: A meta-analysis*, the authors explore “new frontiers” through a timely examination of the widely held assumption that younger workers are more environmentally responsible than older workers, which is particularly relevant in the context of recent demographic, economic, and societal shifts. Specifically, Wiernik et al. (2016) meta-analyze 132 independent correlations and 336 *d*-values based on a total of 4676 professional workers across 22 samples in multiple countries to assess potential age differences in pro-environmental behaviors at work. The authors also draw upon a model of employee green behaviors (Ones and Dilchert, 2012) to examine potential differences among various dimensions including Conserving, Avoiding Harm, Transforming, Influencing Others, and Taking Initiative behaviors. Study results reveal an interesting nuance in the patterns of age-based effects on various dimensions of employee green behaviors. Considered as a whole, however, their findings open “new frontiers” in research and practice by largely debunking age-based stereotypes when it comes to green behaviors at work. The authors conclude that age is likely a “poor proxy” for presumed psychosocial factors (e.g., personality traits, environmental attitudes, or values) in the study of green behavior, or environmental sustainability more broadly, and they suggest that such factors should be measured and directly tested in future research.

## Fulfilling a “Quid Pro Quo”: Implications for Multilevel CSR Research

**FIGURE 1:**
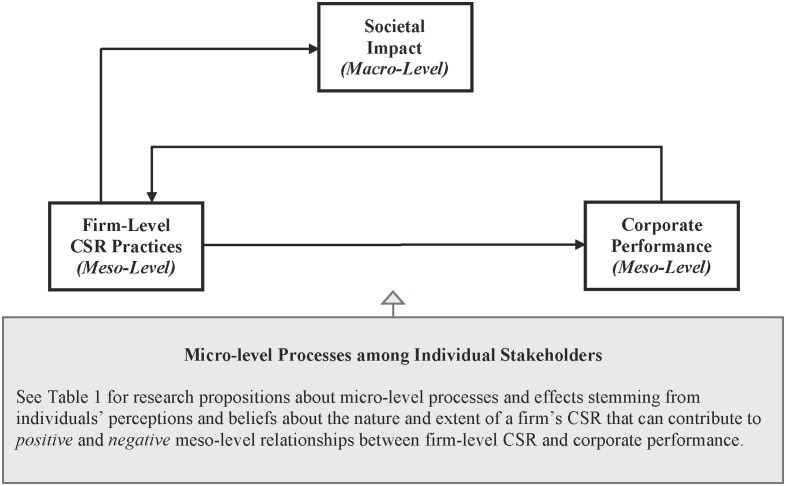
**Insights from Research Topic Articles about Micro-Level Processes that Shape Meso (Firm)-Level Relationships between Corporate Social Responsibility (CSR) Practices and Corporate Performance**.

Having described the “new frontiers” created and explored by the authors of these 12 Research Topic articles, we now leverage insights from these studies in an attempt to fulfill a “quid pro quo” by contributing to some of the meso- and macro-oriented literatures that paved the way for micro-CSR scholarship. In **Figure 1** we present a multilevel model to illustrate ways in which micro-level processes among individual stakeholders can explain variability in meso (firm)-level relationships between CSR practices and corporate performance. We also explore an important implication of these multilevel processes for macro-level societal impact. To be clear, we do not offer this model as a comprehensive multilevel theory of CSR; rather, our objective is far more modest in scope. We offer this model to illustrate ways in which researchers can use insights from micro-CSR studies, such as those that comprise this Research Topic collection, to inform multilevel perspectives that advance CSR theory and research. Supplementing this figure is **Table 1**, where we offer a non-exhaustive list of research propositions pertaining to the multilevel model in **Figure 1**, many of which are intended to illustrate ways in which micro-level insights can inform multilevel research that advances CSR theory and practice. In **Table 1** we cite Research Topic articles for propositions that build on their findings.

**Table 1:** Illustrative research propositions that inform multilevel CSR research, including propositions grounded in findings from research topic articles.

*Illustrative Research Propositions (P) Pertaining to the Multilevel Model in **Figure F1***
• P1: A meaningful amount of the unexplained variability reported in prior research on the meso-level relationships between firm CSR and firm performance is explained by unmeasured micro-level processes that occur among individual job seekers, employees, and other stakeholders. • P2: Some individual-level processes contribute to positive meso-level relationships, and others contribute to negative meso-level relationships; together, these individual-level processes shape the direction and strength of the meso-level relationships. • P3: The virtuous cycle created by the bi-directional causal positive influence between meso-level CSR and corporate performance is strengthened to the extent that companies effectively communicate and manage individual-level beliefs and reactions to their CSR practices. • P4: As firms engage in more strategically managed CSR practices that enhance their corporate performance (including effective communication and management of stakeholder reactions), firms are incentivized to maintain and potentially bolster their subsequent investments in CSR, thereby creating the potential for increasingly higher levels of societal impact. • P5: An individual’s reactions to a firm’s CSR are driven more by their perceptions of the nature and extent of the firm’s CSR compared to the objective nature and extent of the firm’s CSR. • P6: A firm’s internal and external communication about its CSR (or lack thereof) shapes individuals’ perceptions and beliefs about the nature and extent of the firm’s CSR practices.
*Micro-level Processes and Effects that Contribute to Positive Meso-level Relationships*
• P7: CSR enhances a firm’s human capital (and, in turn, firm performance) to the extent job applicants are attracted to working for the firm via their CSR-based inferences about value fit, employee treatment, the work environment, and nature of their prospective coworkers (see Jones et al., 2016). • P8: Responses to CSR that contribute positively to firm performance occur among job seekers and customers to the extent that CSR enhances their trust in the organization, especially when its CSR investments favor their own stakeholder group (see Bridoux et al., 2016). • P9: Employee engagement is enhanced to the extent they perceive their employer’s CSR as authentic, which in turn enhances employee performance and, ultimately, exerts positive effects on firm performance (see Glavas, 2016b). • P10: The performance-oriented behaviors associated with organizational identification exert positive effects on firm performance to the extent that internal and external CSR practices foster identification via internal respect and external prestige, respectively (see Hameed et al., 2016). • P11: Firms that support employee volunteerism can experience employee performance gains caused by improvements in work-related skills through employees’ volunteering experiences (see Jones, 2016). • P12: Firm performance is enhanced via reduced energy costs tied to reduced energy use via leader modeling, prompts, and conservation culture (see Russell et al., 2016).
*Micro-level Processes and Effects that Contribute to Negative Meso-level Relationships*
• P13: Some stakeholders are predisposed to view a firm’s actions negatively, including its more communal pursuits and CSR practices, and their associated reactions exert a negative influence on firm performance (see Unsworth et al., 2016; Voliotis et al., 2016). • P14: Firm performance suffers from negative reactions among job seekers, and presumably other stakeholders, who experience cynicism and skepticism about a given firm’s CSR practices, motives, and claims (see Jones et al., 2016). • P15: Consumers and job seekers can react in ways that negatively influence firm performance to the extent they believe a firm favors other stakeholder groups over their own based on imbalances in its portfolio of CSR practices (see Bridoux et al., 2016). • P16: While the effect of CSR on employee engagement may be more positive when it occurs through other-oriented mechanisms, CSR may result in lower engagement among some employees who are influenced mostly through self-oriented mechanisms, in turn having a negative influence on firm performance (see Glavas, 2016b).

**Figure 1** is centered around the path that represents the effect of firm-level CSR practices on corporate performance—a meso-level relationship that has been the subject of a considerable amount of scholarly debate and research (see Peloza, 2009; Wood, 2010). Among the most frequently studied and (still) hotly debated questions in the broader CSR literature is whether and how CSR practices contribute to or detract from firm performance. Meso-level research has produced mixed results regarding the direction and strength of the relationships between firm-level social/environmental performance and corporate performance (Orlitzky et al., 2003; Margolis et al., 2009; Peloza, 2009; Wood, 2010), with much of this research operationalizing the latter through various indicators of corporate financial performance (e.g., Bansal and Clelland, 2004; Godfrey et al., 2009; Flammer, 2013). Peloza (2009), for example, reviewed 128 empirical studies of this type, and found that the majority reported a positive relationship (59%), but more than one-third reported null or negative relationships.

Researchers have offered multiple explanations for effect size variability in these meso-level relationships, such as stakeholder mismatching between the measures of CSR and firm performance (Wood and Jones, 1995), measurement error and sampling error (Waddock and Graves, 1997), and unaccounted for contingencies (Ullmann, 1985; see also Marom, 2006). Meta-analytic evidence provides some degree of support for all four explanations, while also demonstrating a positive CSR-corporate performance relationship after accounting for statistical and methodological artifacts (Orlitzky et al., 2003). These same authors noted, however, that a considerable amount of unexplained variability in effect sizes across studies still remained, and they urged researchers to identify and test other plausible moderators of CSR-corporate performance relationships. We assert that at least some, and probably much, of this unexplained variability is driven by unmeasured micro-level processes that occur among individual job seekers, employees, and other stakeholders (see Proposition 1, or P1, in **Table 1**) that ultimately lead to both positive *and* negative outcomes that shape the direction and strength of meso-level relationships between firm CSR practices and corporate performance (see P2 in **Table 1**).

We use insights from the Research Topic articles to identify micro-level processes that shape the direction and strength of firm-level effects of CSR on corporate performance. Consistent with longitudinal meta-analytic evidence for what the researchers dubbed a “virtuous cycle” (Orlitzky et al., 2003), **Figure 1** also includes a feedback loop from corporate performance to firm-level CSR to reflect the bi-directional causal influence between the two. That is, as firms engage in more CSR practices their performance tends to increase, and as their performance increases those firms are better positioned and increasingly motivated to allocate additional resources to enhance their CSR practices. The feedback loop represented by this virtuous cycle, we suggest, is strengthened to the extent that companies effectively communicate and manage individual-level beliefs and reactions to their CSR (see P3 in **Table 1**).

**Figure 1** also suggests that this virtuous cycle between meso-level CSR practices and corporate performance has an implication for macro-level societal impact. As firms engage in more strategically valuable CSR practices that enhance their corporate performance, they are incentivized to maintain and even bolster their investments in CSR over time, thereby creating the *potential* for increasingly higher levels of societal impact (see P4 in **Table 1**).

### Micro-level Influences on Meso (Firm)-level CSR Practices

The starting point of the model presented in **Figure 1** is a firm’s CSR practices. Two Research Topic articles provide insights into factors affecting employee behaviors that enhance a firm’s CSR practices relating to its environmental impact. First, employee age appears to have negligible effects on different categories of employee green behaviors and, contrary to pervasive stereotypes, older-aged workers engage in green behaviors slightly more often than their younger-aged counterparts (Wiernik et al., 2016). Moreover, many of these relationships held across 22 organizational contexts in 11 countries. These findings suggest that firms that employ a relatively younger- or older-aged workforce should not be distracted by fretting over whether their employees will embrace the company’s sustainability efforts, nor should managers assume their context is so unique that employee age may indeed matter. For instance, these meta-analytic findings apply to firms that are focused on incremental improvements in reduced energy use (see findings pertaining to employees’ Conserving behaviors) and firms focused on innovating through sustainability initiatives (see findings pertaining to employees’ Transforming and Taking Initiative behaviors). Study findings reported in a second Research Topic article suggest that senior leaders can promote employees’ energy conservation behaviors by demonstrating their own commitment to sustainability through role modeling and communication, and the use of prompts to remind employees to turn off lights, computers, and monitors (Russell et al., 2016). Mixed results were found at the meso-level (i.e., on the firm’s energy conservation), but when unpacked, the results varied depending on behavior at the micro level. Employee influences on meso-level CSR practices were greatest when employees had individual responsibility. For example, for shared resources (e.g., lights), there seemed to be a diffusion of responsibility which led to negligible behavioral change. However, for resources for which employees were responsible (e.g., hard drives, monitors) evidence of longitudinal behavioral change was found. These findings highlight that meso-level effects of CSR on firm performance can be better understood by unpacking some of the micro-level processes and effects involved.

### Micro-level Processes That Contribute to Positive Meso-level CSR-corporate Performance Relationships

We suggested above that some, and probably much, of the unexplained variability in meso-level relationships between firm CSR practices and corporate performance is due to unmeasured micro-level processes that occur among individual job seekers, employees, and other stakeholders. We are not the only, nor the first, researchers to make this assertion: Bauman and Skitka (2012), for example, suggested that the firm-level (meso) relationship between corporate social performance and financial performance is presumable shaped by the effects of CSR on a firm’s ability to attract and retain cooperative and committed employees. Indeed, when one considers that employee attitudes and behaviors are associated with unit-level organizational performance (Koys, 2001), coupled with the growing body of evidence linking CSR to positive employee responses, it is reasonable to expect that some variability in meso-level relationships between CSR and firm performance is attributable to individual-level reactions to CSR. What we uniquely add to prior assertions of this type, however, is a deeper exploration into specific illustrations of pertinent individual-level reactions and processes, and an explicit recognition that these individual-level effects can exert both positive and negative influence on meso-level relationships between CSR and firm performance.

Unfortunately, empirically grounded evidence is severely lacking, as multilevel CSR studies that include analyses of individual-level data are surprisingly rare. Some researchers have studied employee and customer reactions to CSR without collecting any individual-level data from employees or customers, such as by operationalizing CSR, employee retention, and customer satisfaction at the meso-level via survey responses from CEOs (Galbreath, 2010). Other researchers have measured employee attitudes and behaviors at the individual-level and then aggregate that data to create firm-level measures (Chun et al., 2013). However, neither of these two approaches allows researchers to test cross-level effects or interactions. Other multilevel studies have included individual-level measures of employee attitudes and behaviors as well as firm-level measures of socially responsible human resource management (SRHRM) practices, with the latter comprising items like “my company consider employee social performance in performance appraisals” and “my company considers person identity-CSR identity fit in recruitment and selection” (e.g., Shen and Benson, 2016). Notwithstanding the contributions from such studies, the extent to which a company embeds CSR considerations within its HR practices is distinct from the extent to which it engages in CSR practices more broadly, including external CSR practices targeted toward external stakeholders like the natural environment or local community. Other multilevel studies offer insights into multilevel CSR phenomena, but only indirect insights given the absence of CSR measures at any level of analysis. For instance, Parboteeah et al. (2012) tested hypotheses about relationships between cultural dimensions measured at the country-level and people’s propensity to support sustainability initiatives at the individual-level, but none of the measures used in this study contained references CSR, sustainable business, or company practices of any type.

One recent study, however, tested relationships among meso-level CSR and micro-level employee attitudes. Suh (2016) measured meso-level CSR (measured via an independent index), meso-level communication (aggregated across individual-level survey responses), and individual-level employee attitudes and demographics. Results showed that firm-level CSR predicted employee job satisfaction and affective commitment, mediated by employee perceptions of their relational social capital (e.g., the quality of their work relationships). Although these findings do not provide evidence about whether and the extent to which individual-level reactions to CSR can explain variability in meso-level relationships between CSR and firm performance, Suh’s (2016) methodology offers guidance to researchers interested in this topic. Extending this design approach, we believe there is considerable value in measuring CSR at both the meso-level (i.e., using an objective measure of a firm’s CSR) and the individual-level (i.e., using a perceptual measure of stakeholder beliefs about a firm’s CSR). By including both types of CSR measures in multilevel research, scholars can begin to explore practically important research questions, such as investigations of the factors that explain convergence and divergence between a firm’s actual CSR practices and how they are perceived by individual stakeholders.

As several Research Topic articles highlight, micro-CSR research shows that individual-level stakeholder reactions to CSR are driven, at least in part, by each individual’s perceptions and beliefs about the nature and extent of a firm’s CSR practices (e.g., Glavas, 2016b; Hameed et al., 2016; Jones et al., 2016; Seivwright and Unsworth, 2016; Voliotis et al., 2016). Scholars have emphasized the importance of focusing on individuals’ *perceptions* of CSR when attempting to understand individual reactions to CSR (e.g., El Akremi et al., 2015; Glavas, 2016a), and we assert that an individual’s reactions to CSR are driven less by CSR practices as they objectively exist, and more by how that individual perceives them to exist (see P5 in **Table 1**). As such, most types of stakeholder reactions are bounded by the extent to which a firm can effectively communicate about its CSR practices to individuals, and how those individuals perceive and interpret those practices (see P6 in **Table 1**). While beyond the scope of this article, effective CSR communication is critically important to realizing its potential value to various stakeholders—including shareholders and owners. We direct readers to Du et al. (2010) for an excellent discussion of the importance of communicating about CSR commitment, impact, fit, motives, and other factors.

As reflected in **Figure 1**, individual-level processes can exert positive *and* negative influence on the direction and strength of meso-level relationships between CSR and corporate performance, which we illustrate through research propositions presented in **Table 1** that build on findings from some of the Research Topic articles. Our intent is not to review the nuanced findings from the Research Topic articles, but to focus on the overall micro-level processes they highlight. For instance, personality and values affect stakeholder reactions to CSR, such that individual stakeholders tend to respond more positively when they have a stronger calling orientation (Hameed et al., 2016) and other orientation (Bridoux et al., 2016); while important, for the present purposes we focus on the broader individual-level processes demonstrated by the Research Topic articles.

Starting with reactions to CSR among prospective employees, findings from Jones et al. (2016) suggest that individual-level processes among job seekers likely influence firm-level effects of CSR on corporate performance. CSR can be leveraged to attract more applicants, thereby increasing the size of the applicant pool. In turn, by improving a firm’s chances of hiring talented employees (Ployhart, 2006; Breaugh, 2008), CSR practices can enhance the quality of the firm’s human capital. Variability in the extent to which firms leverage their CSR during employee recruitment in accordance with these micro-level processes, we assert, can explain some of the variability in the meso-level relationships between firm-level CSR practices and corporate performance. That is, the meso-level effect of CSR on corporate performance will be stronger among firms that communicate their CSR practices in ways that come to the attention of job seekers, and that lead job seekers to infer higher levels of perceived value fit, favorable employee treatment, a positive work environment, and desirable characteristics and values among their prospective coworkers (see P7 in **Table 1**). Conversely, the meso-level effect of CSR on corporate performance will be weaker to the extent that the messages job seekers receive about the firm’s CSR do not lead them to make such inferences, especially to the extent that job seekers remain unaware of the firm’s CSR practices in the first place.

Bridoux et al.’s (2016) study of stakeholder reactions to CSR tradeoffs also focused on reactions among job seekers, as well reactions among potential customers. Their findings suggest that these two stakeholder groups tend to respond positively to CSR to the extent it enhances their trust in the organization, especially when the firm’s investments in CSR favors their own stakeholder group (see P8 in **Table 1**). Accordingly, corporate leaders should strive to allocate sufficient resources to CSR in a manner that targets the stakeholder groups on which the firm strongly depends, and they should communicate about the firm’s CSR in ways that demonstrate trustworthiness. In doing so, a firm can leverage its CSR to attract talented workers and enhance customer loyalty, and ultimately improve its human capital and market share. To operationalize CSR tradeoffs and assess such multilevel processes and effects, researchers could collect objective indicators of corporate performance and firm-level investments in multiple types of CSR practices, which is a legally mandated reporting requirement in some countries (e.g., Pakistan). Researchers could also collect individual-level survey data to measure trust and trustworthiness to assess whether firm-level CSR tradeoffs have effects on individual-level trust among important stakeholder groups that might ultimately shape meso-level CSR-corporate performance relationships.

Most of the other Research Topic articles focus on responses to CSR among incumbent employees, such as engagement and other indicators of employee commitment and performance that are known to contribute to corporate performance. Glavas (2016b) showed that employees have higher levels of engagement—an important motivator of employee performance (Rich et al., 2010)—when their employer’s overall CSR practices allow them to demonstrate authenticity by bringing more aspects of their whole selves to work (see P9 in **Table 1**; and see Seivwright and Unsworth, 2016 for insights about the interplay between employees’ experience of CSR and the meaning they find through their work). Hameed et al. (2016) demonstrated that internal and external CSR practices can foster employees’ sense of internal respect and their perception of their employer’s external prestige, which ultimately enhanced employees’ organizational identification, which is known to motivate employee commitment and performance (see P10 in **Table 1**). Jones (2016) reported evidence that employees who participate in corporate volunteering can develop work-related skills that relate to job performance, such as teamwork, project management, time management, public speaking, and leadership skills (see P11 in **Table 1**). Given that firms allocate meaningful resources toward training and professional development, these findings raise the possibility that companies can achieve some of the same ends through alternative investments in community-focused CSR practices that may simultaneously create additional value for the firm through reputation enhancement. Russell et al.’s (2016) intervention study showed that employees were encouraged to reduce their energy use at work when managers role modeled commitment to environmental practices, provided prompts and reminders, and communicated to create a culture of energy conservation (see P12 in **Table 1**).

These articles illustrate positive employee responses to multiple types of CSR practices directed toward different stakeholder groups. Overall, these findings suggest that positive meso-level relationships between firm CSR and corporate performance are enhanced by micro-level processes among individual stakeholders through which CSR practices: (1) become known to stakeholders, (2) attract job seekers by informing their inferences about value fit and other matters, (3) create trust that promotes desirable reactions among job seekers and customers, and (4) fosters positive employee attitudes and behaviors such as employee engagement, organizational identification, work-related skills, and reduced energy use at work. To the extent a firm’s CSR practices are managed and perceived in ways that foster these and other micro-level processes, the meso-level relationship between a firm’s CSR practices and corporate performance will be increasingly positive and robust.

### Micro-level Processes That Contribute to Negative Meso-level CSR-corporate Performance Relationships

Almost all CSR practices require short- and long-term investments of firm resources, and firms also incur associated opportunity costs that affect its corporate performance. In this context, when CSR practices are not managed or communicated well, any potentially positive returns from CSR are diminished. Moreover, research propositions 13 through 16 (see **Table 1**) highlight that some stakeholders can respond *negatively* to CSR practices, which can ultimately weaken the otherwise positive meso-level effects of CSR on corporate performance.

To date, the vast majority of micro-CSR studies have documented positive stakeholder responses to CSR practices that plausibly contribute to positive meso-level relationships with corporate performance. The authors of four Research Topic articles, however, conceptually explore and empirically demonstrate that negative individual-level stakeholder responses to CSR practices also occur (Bridoux et al., 2016; Glavas, 2016b; Jones et al., 2016; Voliotis et al., 2016). Voliotis et al. (2016) developed a model of stakeholder perceptions and reactions to companies that engage in socially responsible vs. socially irresponsible practices, and we believe some of their arguments can be extended to contexts in which individual stakeholders can come to very different conclusions about a single firm’s CSR practices. Voliotis et al. (2016) noted that people tend to stereotype for-profit companies as being generally unconcerned about communal pursuits and contributing to society beyond providing economic opportunities (e.g., hiring employees) and meeting consumer needs (e.g., selling products and services). Relatedly, Unsworth et al. (2016) reported that survey respondents identified ‘industry/companies’ as the actor who bears the greatest responsibility for climate change. As such, some internal and external stakeholders may be predisposed to perceiving any corporate activity as inherently irresponsible, priming them to react with mistrust and suspicion toward a given firm’s CSR practices (see P13 in **Table 1**). Indeed, research on CSR attributions shows that consumers and employees can hold widely different views about a company’s motives for its CSR initiatives, resulting in correspondingly positive and negative reactions to the company’s CSR practices (e.g., Vlachos et al., 2010). Glavas (2016b) found that when CSR is extra-role (e.g., volunteering) it can lead to negative effects on employee engagement. For a smaller number of hours in volunteering (i.e., 1–12 h per year), there was a positive effect on employees but as the hours increased, employees felt role strain and CSR became a burden. These findings suggest that the impact of CSR on employees varies depending on how it is embedded in their jobs (i.e., whether CSR-related behaviors are in-role vs. extra-role).

As Aguinis and Glavas (2013) suggested, a company often implements its CSR practices in various ways and to varying degrees throughout its different divisions and functional areas. As such, while some employees might be exposed to substantive value-creating CSR practices that clearly benefit multiple stakeholders, other employees within the same firm might only be exposed to largely symbolic and superficial CSR practices that they view with cynicism and skepticism (Willness and Jones, 2013). Such cynicism and skepticism about a firm’s CSR practices was uncovered in one Research Topic article focusing on job seeker reactions to CSR. Specifically, Jones et al. (2016) reported meaningful differences in people’s interpretations of a single firm’s community-focused and environmentally sustainable practices. While about two-thirds of the participants claimed they were more attracted to the employer because of its CSR practices, the remaining third reported being largely unaffected by the CSR information—including some people who described cynicism and skepticism about CSR. This study was conducted in an employee recruitment context, and other research suggests that cynicism and skepticism about CSR also exists among consumers (Du et al., 2010). Accordingly, an important boundary condition that likely weakens the potentially positive meso-level relationships between CSR and corporate performance is the extent to which job seekers, consumers, and other individual stakeholders are cynical or skeptical about a firm’s CSR practices. When pervasive, these negative stakeholder reactions might result in a negative effect of firm-level CSR on corporate performance (see P14 in **Table 1**). Specific factors contributing to such cynicism and skepticism are discussed in the Jones et al. (2016) Research Topic article and elsewhere (e.g., Du et al., 2010; Vlachos et al., 2010; Willness and Jones, 2013).

In another Research Topic article, Bridoux et al. (2016) documented another context in which some stakeholders react negatively to a firm’s practices: CSR tradeoffs. Their overall pattern of results suggests that consumers and job seekers can react negatively when they believe a company favors other stakeholder groups over their own based on its differential investments and focus in its portfolio of CSR practices (see P15 in **Table 1**). Another Research Topic article uncovered potentially negative responses among a different stakeholder group: a firm’s employees. Glavas (2016b) tested two mediators of the relationship between a firm’s overall CSR practices and employee engagement. His findings suggest that the effects of CSR through other-oriented mechanisms tend to result in more positive employee reactions compared to effects through more self-oriented mechanisms (see P16 in **Table 1**).

In our review of insights from Research Topic articles in this and the preceding subsection, we illustrated ways in which micro-level processes can lead to positive and negative reactions to CSR among a firm’s internal and external stakeholders. While little evidence exists to estimate the extent of their influence, we speculate that the net effect of unmeasured positive and negative stakeholder reactions may be the primary “culprit” underlying the mixed results found in meso-level research on firm CSR practices and corporate performance (Peloza, 2009; Wood, 2010). We hope meso-oriented CSR scholars find value in our examples of pertinent micro-level processes that can inform multilevel research that advances the CSR literature. We now turn to another important topic in CSR research that has received relatively little attention, and that lends itself to multilevel theory and research that incorporates processes at the micro-, meso-, and macro-levels of analysis.

### Micro-level Processes and Meso-level Effects of CSR on Macro-level Societal Impact

There is a pervasive, and sometimes unfounded, belief that corporate-level decisions to invest in CSR initiatives rest entirely on the presence of a compelling “business case” (Hafenbrädl and Waeger, 2016). We believe that, in reality, corporate decisions to invest in CSR are often more nuanced than most people probably assume. CSR allocation decisions are not made by “corporations,” but by business leaders who, like most other people, have multiple motives underlying most of the things they do, including their decisions to pursue a CSR agenda (Aguilera et al., 2007). Many business leaders with whom we (the authors) have interacted demonstrate some degree of genuine concern and care for societal impact, and especially for the stakeholders directly affected by the company’s operations and practices; and other business leaders with whom we’ve interacted appear to be driven more by instrumental motives linked to risk mitigation and short-term profit.

We have little reason to doubt that the “business case” for CSR plays a major role in how CSR initiatives are resourced and managed in most for-profit companies, and there remains substantive debate about the role of business in society and the extent to which companies *should* engage in CSR—or not (e.g., Margolis and Walsh, 2003; Orlitzky, 2015). Our contention, however, is that this largely unchallenged assumption is so widely held among CSR scholars that they have focused much of their research energy on the meso-level relationship between firm CSR practices and corporate financial performance (Aguinis and Glavas, 2012), and they have done so at the expense of understanding the actual impact of CSR practices on external stakeholders and society at large (Margolis and Walsh, 2003; Wood, 2010). Scholars have called for multilevel theories and studies of CSR (e.g., Glavas, 2016a), and we urge researchers to include macro-level societal impact in these efforts. To borrow a phrase from Aguilera et al. (2007), we as a scientific community need to bring the “S” (i.e., society) back into CSR research.

Some of the 12 Research Topic articles specifically point to micro-level processes that have implications for societal impact. For instance, Voliotis et al. (2016) focus on how stakeholders perceive and react to companies that engage in socially responsible and socially irresponsible practices; the Jones (2016) article suggests that non-profits can leverage the increasing prevalence of corporate volunteerism programs to better achieve their missions by designing opportunities for employee volunteers to improve their work-related skills and, by extension, attract more interest from corporate partners; and the Russell et al. (2016) study provides practical guidance to firms seeking to reduce their energy consumption through individual employee behavior. The findings from most of the Research Topic articles, however, do not easily translate into insights for macro-level CSR research on the influence of broader economic, institutional, and societal contexts *per se* (e.g., Matten and Moon, 2008; Frynas and Stephens, 2015). In perhaps one exception, Unsworth et al.’s (2016) study illuminates stakeholder reactions that can inform regulatory policy about environmental practices, such as adhering to toxic waste disposal bylaws or emissions reporting requirements.

The multilevel model shown in **Figure 1**, however, does have a more general implication pertaining to the role of individual-level reactions in the effects of firm-level CSR practices on macro-level societal impact. We acknowledge that CSR practices, no matter the intentions and motives that prompted them, do not necessarily create positive societal impact. For instance, CSR practices likely have unintended consequences that can create negative societal impact. Moreover, as companies increase their investments in CSR over time, it might reduce societal support for government programs as a means to address societal ills. We think it is likely, however, that the net effect of companies’ CSR programs is generally positive for employees, consumers, communities, and the natural environment.

We believe that insights about micro-level processes can meaningfully inform the strategic management of CSR practices to generate greater financial returns through positive stakeholder reactions. As firms reap economic benefits from well-managed CSR practices, they become increasingly motivated and able to invest additional resources to expand their CSR practices. To the extent this “virtuous cycle” between CSR and corporate performance (Orlitzky et al., 2003) leads a firm to increase its CSR investments over time, the potential result is increasingly greater social value and macro-level societal impact created by the firm’s CSR practices. In this way, insights about micro-level stakeholder reactions that inform how CSR practices can be managed to create stronger financial returns may indirectly promote macro-level societal impact.

## Concluding Comments

In recent years, there has been a veritable explosion of micro-CSR research, and a rising recognition of the important role these processes play in affecting CSR practices and corporate performance. Through co-editing a Research Topic collection, we sought to facilitate additional advancements in micro-CSR theory and research, and herein we described some of the “new frontiers” these articles explored and created. We also sought to fulfill a “quid pro quo” by drawing on insights from the micro-oriented Research Topic articles to help explain variability in meso (firm)-level relationships between CSR practices and corporate performance. Our hope is that we have helped to build stronger bridges that can—and should—exist between micro-, meso-, and macro-oriented research on CSR.

As Frederick (2016: 2) stated “Now, a new CSR stage—CSR5: Sustainability (2000–2050)—began with the opening of the new millennium…. I believe that an integrated, holistic solution will be sought, and hopefully found, by a coalition of ‘policy-makers’ and ‘people.’ I invite and urge you to read the papers in this collection to discover how the ‘Policy to People’ goal can be approached and eventually attained.”

## Author Contributions

All authors contributed equally to this work.

## Conflict of Interest Statement

The authors declare that the research was conducted in the absence of any commercial or financial relationships that could be construed as a potential conflict of interest.

## Footnotes

1 We adopt the micro-, meso-, and macro- labels to draw attention to these distinctions and encourage micro-CSR scholars to more clearly distinguish between meso- and macro-level phenomena, and to facilitate understanding among non-micro oriented readers who may be more familiar with the labels adopted herein. As observed by one reviewer, however, micro individual-level research need not be psychological in nature, and group-level research can encompass psychological constructs (e.g., research on group-level climates).
